# Differences in Chemical Components and Antioxidant Ability Analysis of *Pseudostellaria heterophylla* from Multiple Origins

**DOI:** 10.3390/ijms27073139

**Published:** 2026-03-30

**Authors:** Wujun Zhang, Xiaolan Xu, Jingying Chen, Yunqing Zhao, Baocai Liu, Yingzhen Huang

**Affiliations:** 1 Institute of Crop Research, Fujian Academy of Agricultural Sciences, Fuzhou 350013, China; zhangwujun@faas.cn (W.Z.); zhaoyunqing@faas.cn (Y.Z.); huangyingzhen@faas.cn (Y.H.); 2 College of Bee Science and Biomedicine, Fujian Agriculture and Forestry University, Fuzhou 350002, China; xlxufz@126.com

**Keywords:** *Pseudostellaria heterophylla*, producing area variation, untargeted metabolomics, differential metabolites, antioxidant activity

## Abstract

Taizishen is a traditional Chinese medicinal herb derived from the dried tuberous roots of *Pseudostellaria heterophylla*. This study investigated the compositional variation of Taizishen from main producing (MP) and non-main producing (NP) areas across five Chinese provinces. Analysis of total saponins, flavonoids, and heterophyllin B showed the highest contents in Jurong samples, followed by Zherong. Untargeted metabolomics identified 651 metabolites in all samples. Principal component analysis revealed a distinct metabolic profile for the sample from Zherong, which differed significantly from other MP areas, showing 32 consistently upregulated (e.g., amino acids, terpenes) and 25 downregulated metabolites (e.g., lipids, alkaloids). Notably, key differential metabolites such as fraxetin and ethyl caffeate were enriched in Zherong samples. The number of differential metabolites between MP and NP areas varied by province. Antioxidant activity also varied regionally, being highest in the sample from Jurong and weakest in the sample from Duyun. Correlation analysis indicated this activity was not linked solely to flavonoid or saponin content, suggesting a synergistic effect of multiple components. In addition, Zherong samples exhibited unique accumulation patterns for amino acids, sugars, and lipids. The significant metabolic and bioactivity variations highlight the need for a comprehensive, metabolomics-informed quality evaluation system for Taizishen.

## 1. Introduction

Taizishen, the dried tuberous root of *Pseudostellaria heterophylla* (Miq.) Pax ex Hoffm., is widely distributed across many regions of China [1]. Among them, Fujian, Jiangsu, Anhui, Shandong, and Guizhou are recognized as the major cultivation areas [1,2]. Previous studies have demonstrated that the main chemical constituents of Taizishen comprise cyclic peptides, saponins, volatile oils, sugars and glycosides, fatty acids and esters, amino acids, and trace elements [3]. Pharmacological investigations have revealed that Taizishen exhibits a broad range of biological activities, including the amelioration of chronic heart failure, anti-inflammatory effects, immunomodulatory activity, and hypoglycemic properties. Its antioxidant capacity is regarded as one of the fundamental mechanisms underlying these health-promoting properties [4,5,6,7].

The place of origin significantly influences the growth, development, and accumulation of active constituents in traditional Chinese medicinal materials through factors such as climatic conditions, soil characteristics, topography, and the ecological environment, thereby determining their quality [8,9]. Owing to the wide geographical distribution and diverse cultivation environments of Taizishen, substantial ecological and climatic heterogeneity exists among different production areas. Such environmental variability is likely to contribute to pronounced differences in chemical composition and bioactivity [10,11]. Currently, the medicinal quality of Taizishen is primarily evaluated based on the quantification of selected constituents, including cyclic peptides, saponins, polysaccharides, and nucleosides [12,13,14]. However, Taizishen exhibits a highly complex chemical profile, and its pharmacologically active components have not yet been fully characterized. Moreover, its chemical composition can vary markedly in response to differences in producing areas and agricultural practices [15,16,17,18], which may ultimately result in variability in pharmacological efficacy.

Therefore, a comprehensive quality assessment method is crucial. Currently, assessing the medicinal quality of Taizishen based on a single component or a limited number of markers is insufficient to comprehensively reflect its overall quality. Some analytical techniques, including HPLC fingerprint analysis, have been employed to characterize the chemical constituents of Taizishen from different geographical origins and germplasms [19]. In parallel, multivariate statistical approaches, such as principal component analysis (PCA), partial least squares discriminant analysis (PLS-DA) and orthogonal PLS-DA (OPLS-DA), have been widely applied to identify representative or discriminative chemical markers [20,21,22]. With the increasing market demand for Taizishen, its cultivation has expanded into numerous non-native regions, resulting in a further diversification of producing areas. Nevertheless, comparative studies systematically evaluating the chemical characteristics of Taizishen from main producing (MP) areas and non-main producing (NP) areas remain limited.

In recent years, metabolomics has been widely applied to investigate the geo-authenticity of traditional Chinese medicinal materials, enabling the characterization of differential components among samples from distinct geographical origins [23,24]. For example, metabolomics combined with multiple discriminant models has been used to distinguish the geographical origin and growth patterns of *Astragalus* species, revealing that secondary metabolites, such as astragaloside IV, calycosin-7-O-β-D-glucoside, and ononin, played critical roles in identification [25]. To comprehensively evaluate the impact of geographical origin on Taizishen quality, the present study employed an untargeted metabolomics approach. We systematically compared the metabolite profiles of samples collected from ten cultivation sites across five provinces in China, including Fujian, Anhui, Guizhou, Jiangsu, and Shandong. Encompassing both traditional main producing (MP) areas (Zherong, Xuancheng, Shibing, Jurong, and Linmu) and non-main producing (NP) areas. The contents of major active components, including total flavonoids, total saponins, and heterophyllin B, as well as the total antioxidant capacity, were also simultaneously determined. Comparative analyses were conducted to elucidate differences in metabolite composition and antioxidant activity between samples from MP and NP areas within the same province. This study provides a comprehensive metabolomic characterization and analysis of major active components of Taizishen, offering fundamental data to support the evaluation of its quality and pharmacological activity.

## 2. Results

### 2.1. Determination of Flavonoid and Saponin Contents

**Figure 1:**
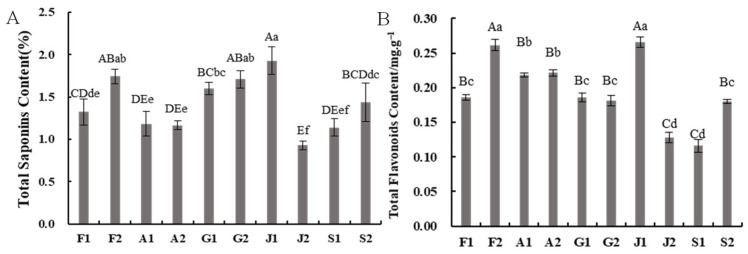
Total saponin content (**A**) and total flavonoid content (**B**) of Taizishen from different producing areas. Values are mean ± SD (*n* = 3). Bars with different letters are significantly different (uppercase, *p* < 0.01; lowercase, *p* < 0.05; Duncan’s test). Sample codes: F1 (Fuan, Fujian, NP); F2 (Zherong, Fujian, MP); A1 (Guangde, Anhui, NP); A2 (Xuancheng, Anhui, MP); G1 (Shibing, Guizhou, MP); G2 (Duyun, Guizhou, NP); J1 (Jurong, Jiangsu, MP); J2 (Ganyu, Jiangsu, NP); S1 (Changyi, Shandong, NP); S2 (Linshu, Shandong, MP). MP, main producing area; NP, non-main producing area.

The contents of saponins and flavonoids in Taizishen from different producing areas showed significant variation (Figure 1). The highest saponin concentrations were found in samples J1, F2, and G2, whereas the lowest levels were in J2 and S1. Similarly, total flavonoid content varied considerably across samples from different regions. The highest levels were observed in samples J1 and F2, followed by A1 and A2, while the lowest concentrations were detected in samples J2 and S1. The results indicated no significant differences in total flavonoid and saponin contents between MP and NP areas in Guizhou and Anhui provinces. However, in the MP areas of the remaining three provinces (Fujian, Jiangsu, and Shandong), the contents of both total flavonoids and saponins were significantly higher compared with their NP areas.

### 2.2. Heterophyllin B Content in Taizishen from Different Producing Areas

**Figure 2:**
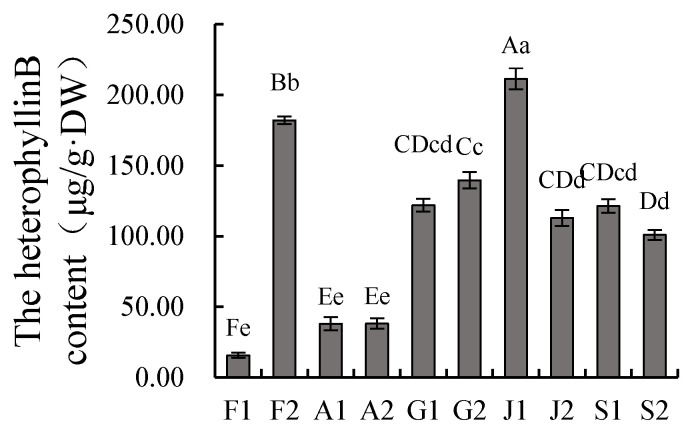
Heterophyllin B content in *P. heterophylla* from different producing areas. Values are mean ± SD (*n* = 3). Bars with different letters are significantly different (uppercase, *p* < 0.01; lowercase, *p* < 0.05; Duncan’s test). Sample codes: F1 (Fuan, Fujian, NP); F2 (Zherong, Fujian, MP); A1 (Guangde, Anhui, NP); A2 (Xuancheng, Anhui, MP); G1 (Shibing, Guizhou, MP); G2 (Duyun, Guizhou, NP); J1 (Jurong, Jiangsu, MP); J2 (Ganyu, Jiangsu, NP); S1 (Changyi, Shandong, NP); S2 (Linshu, Shandong, MP). MP, main producing area; NP, non-main producing area.

The heterophyllin B content in Taizishen from different producing areas was quantified using HPLC (Figure 2). The highest concentration was observed in sample J1 (211.47 μg/g DW), followed by F2 (182.08 μg/g DW) and the samples from the four producing areas in Guizhou and Shandong provinces. In contrast, the heterophyllin B content in samples F1, A1, and A2 was significantly lower than that in the other producing areas.

### 2.3. Results ofMetabolomics Analysis

#### 2.3.1. LC-QTOF-MS Detection Results

**Figure 3:**
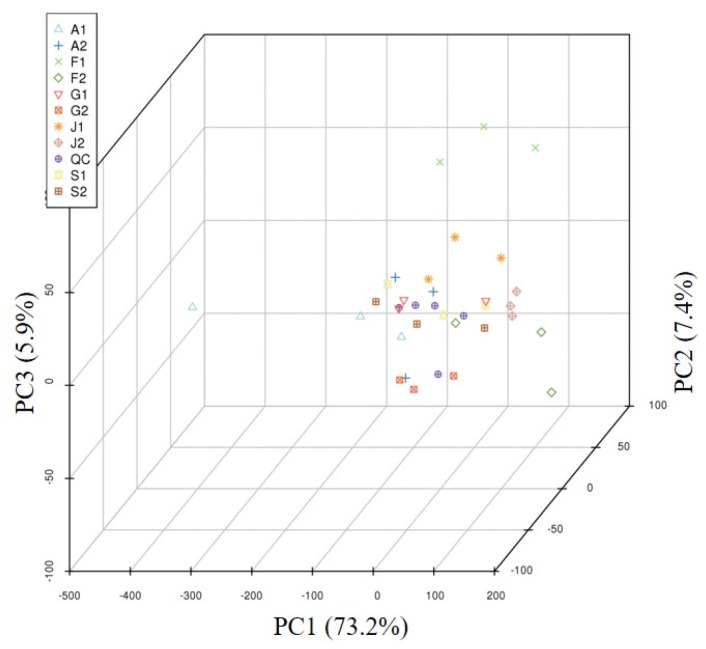
Two-dimensional plot of PCA for metabolites in *P. heterophylla* from different producing areas. Sample codes: F1 (Fuan, Fujian, NP); F2 (Zherong, Fujian, MP); A1 (Guangde, Anhui, NP); A2 (Xuanchen, Anhui, MP); G1 (Shibin, Guizhou, MP); G2 (Duyun, Guizhou, NP); J1 (Jurong, Jiangsu, MP); J2 (Ganyu, Jiangsu, NP); S1 (Changyi, Shandong, NP); S2 (Linmu, Shandong, MP). MP, main producing area; NP, non-main producing area. QC: quality control samples.

A total of 651 metabolites were detected in Taizishen from different regions (Supplementary Table S1A). These metabolites included, but were not limited to, amino acids and their derivatives, flavonoids, lipids, nucleotides and their derivatives (Supplementary Table S1B). PCA results showed that samples from Fujian province could be clearly separated from those from other provinces based on the detected metabolites, while samples from the remaining provinces could not be distinctly differentiated from each other (Figure 3).

To further analysis the differences between these regions, OPLS-DA was applied. The results indicated that the samples from different regions could be effectively separated using the OPLS-DA model (Supplementary Figure S1). The model parameters for pairwise comparisons between regions are presented in Supplementary Table S1C. The R^2^Y (cum) and Q^2^ (cum) values for all comparison groups were exceeded 0.5, indicating the reliability of the model for differential metabolite analysis.

#### 2.3.2. Differential Metabolite Analysis

##### Comparison Between MP and NP Areas Within Each Province

**Figure 4:**
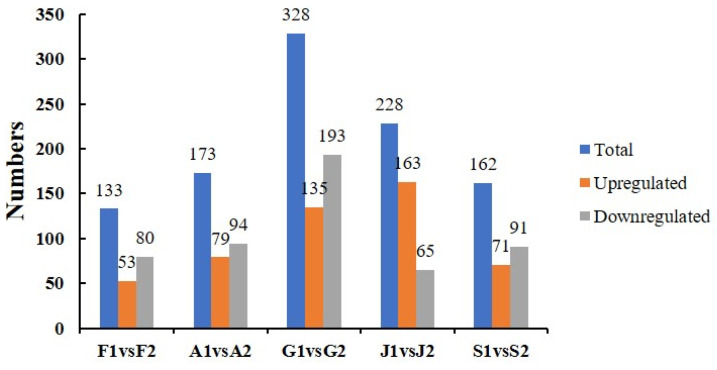
Number of differential metabolites in *P. heterophylla* from MP and NP areas within the same province. Sample codes: F1 (Fuan, Fujian, NP); F2 (Zherong, Fujian, MP); A1 (Guangde, Anhui, NP); A2 (Xuanchen, Anhui, MP); G1 (Shibin, Guizhou, MP); G2 (Duyun, Guizhou, NP); J1 (Jurong, Jiangsu, MP); J2 (Ganyu, Jiangsu, NP); S1 (Changyi, Shandong, NP); S2 (Linmu, Shandong, MP). MP, main producing area; NP, non-main producing area.

The number of significantly different metabolites between the main and non-main producing areas of Taizishen within each province is shown in Figure 4, and the detailed differential metabolites are listed in Supplementary Tables S2A–E. The results revealed that, except for Jiangsu Province, the NP areas in the other four provinces generally exhibited a higher number of downregulated metabolites compared totheir respective MP areas. Among all provinces, Guizhou showed the largest number of differential metabolites, while Fujianhad the fewest.

A comparison was made between the two producing areas within each province based on the VIP values of the metabolites derived from the OPLS-DA analysis. The top 20 differential metabolites for each province are listed in Supplementary Table S2F. Compared to Zherong, Fuan exhibited 3 upregulated metabolites and 17 downregulated metabolites, predominantly including flavonoids, phenylpropanoids, amino acids, and small molecules. In the comparison between Xuancheng and Guangde, Guangde showed 2 upregulated and 18 downregulated metabolites, with the downregulated ones consisting mainly of alkaloids, amino acids and their derivatives, and organic acids. When comparing Shibing and Duyun, Duyun showed 2 upregulated and 18 downregulated metabolites, with the downregulated ones consisting mainly of organic acids and fatty acid derivatives. In contrast, compared to Jurong, Ganyu exhibited 18 upregulated metabolites and only 2 downregulated metabolites. In the comparison of Linmu and Changyi, Changyi showed 12 upregulated metabolites (including organic acids, flavonoids, lipids, and others) and 8 downregulated metabolites, the majority of which were flavonoids.

These results indicate that, except for Jiangsu Province, the top 20 differential metabolites (with the highest VIP values) in the NP areas of the other four provinces were predominantly downregulated compared to their corresponding MP areas.

##### Comparison Between Zherong and Other Main Production Areas

**Figure 5:**
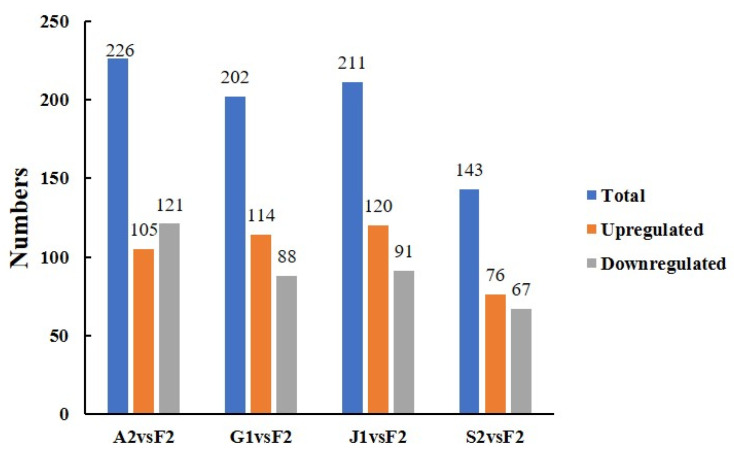
Number of differential metabolites in *P. heterophylla* from other MP areas compared to the Zherong area. Sample codes: F2 (Zherong, Fujian, MP); A2 (Xuanchen, Anhui, MP); G1 (Shibin, Guizhou, MP); J1 (Jurong, Jiangsu, MP); S2 (Linmu, Shandong, MP). MP, main producing area.

The number of significantly different metabolites between the Zherong area and the MP areas of Taizishen in other provinces is shown in Figure 5. Overall, except for Xuancheng, comparisons with Zherong showed a predominance of upregulated metabolites in the other MP areas. The largest number of differential metabolites was observed between Xuancheng and Zherong, while the fewest were found between Linmu and Zherong.

The top 20 differential metabolites (ranked by VIP value) from the OPLS-DA models comparing the main production areas in each province with Zherong are listed in Supplementary Table S3E. Compared to Zherong, Xuancheng exhibited 8 upregulated metabolites, mainly organic acids and alkaloids, and 12 downregulated metabolites, primarily flavonoids, terpenes, and phenolic derivatives. Shibingshowed 16 upregulated metabolites, predominantly fatty acid oxidation derivatives, and 4 downregulated metabolites. Jurong exhibited 14 upregulated metabolites, including fatty acids and their derivatives, and alkaloids, along with 6 downregulated metabolites. Linmu showed 16 upregulated metabolites, mainly flavonoids and alkaloids, and 4 downregulated metabolites.

A comparison between the F2 sample (Zherong) and the other four MP areas revealed 57 significantly upregulated or downregulated differential metabolites (Supplementary Table S4). Among these, 32 metabolites were more abundant in F2. These upregulated metabolites included amino acids and derivatives (18.8%; e.g., γ-aminobutyric acid, γ-glutamyl-leucine, histidine, leucine, L-threonine, and phenylalanine), terpenoids (18.8%; e.g., Bruceine D), and saccharides (9.38%;e.g, raffinose, trehalose). (Supplementary Table S4A). Notably, fraxetin was the most significantly down-regulated compound in the other regions compared to Zherong (average log_2_FC = −3.21), indicating its content was markedly higher in Zherong. Similarly, ethyl caffeate showed a higher level in Zherong (average log_2_FC = −2.54 in other regions). These compounds may serve as key chemical markers for distinguishing the Zherong origin. Conversely, 25 metabolites were less abundant in F2 compared to the other four MP areas, predominantly lipids (56%), alkaloids (16%), and terpenoids (12%) (Supplementary Table S4B), suggesting a more active lipid oxidation metabolic pathway in the other MP areas.

### 2.4. Analysis of Antioxidant Capacity

**Figure 6:**
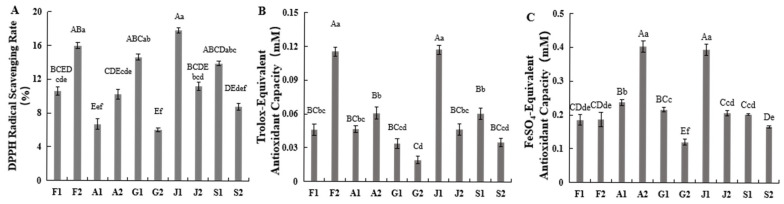
Analysis of antioxidant capacity of *P. heterophylla* from different production areas. (**A**) DPPH Free Radical Scavenging Rate, (**B**) ABTS Free Radical Scavenging Rate, (**C**) Iron Ion Reducing Ability. Values are mean ± SD (*n* = 3). Bars with different letters are significantly different (uppercase, *p* < 0.01; lowercase, *p* < 0.05; Duncan’s test). Sample codes: F1 (Fuan, Fujian, NP); F2 (Zherong, Fujian, MP); A1 (Guangde, Anhui, NP); A2 (Xuancheng, Anhui, MP); G1 (Shibing, Guizhou, MP); G2 (Duyun, Guizhou, NP); J1 (Jurong, Jiangsu, MP); J2 (Ganyu, Jiangsu, NP); S1 (Changyi, Shandong, NP); S2 (Linshu, Shandong, MP). MP, main producing area; NP, non-main producing area.

As shown in Figure 6, samples J1 and F2 exhibited the highest DPPH· scavenging rates, followed by samples G1, S1, F1 and J2, while samples A1 and G2 showed the lowest. Both the ABTS and FRAP assays consistently demonstrated that samples J1 and F2 possessed the strongest antioxidant activity, whereas G2 displayed the weakest. Except for Shandong Province, the MP areas in the other provinces showed stronger antioxidant activity compared to their corresponding NP areas. Correlation analysis indicated that neither saponins nor heterophyllin B correlated with the three types of antioxidant activities, while total flavonoids exhibited a moderate correlation with both ABTS and IPTZ, with correlation coefficients of 0.55 and 0.60, respectively.

## 3. Discussion

Under the influence of both ecological environment and cultivation practices, geo-authentic medicinal materials develop unique quality characteristics that distinguish them from similar medicinal materials produced in other regions, manifesting in their morphological traits, chemical composition, pharmacological activity, and clinical efficacy [26,27]. This study employed a systematic analysis of the chemical composition profile of Taizishen, providing a comprehensive assessment of its intrinsic quality. While our PCA results confirm the distinct chemical profile of samples from Zherong county—consistent with earlier fingerprinting studies noting low correlation coefficients between Fujian and other provinces [19], the present analysis delivers a deeper mechanistic interpretation. We identify a specific metabolic signature for Taizishen in Zherong characterized by the coordinated upregulation of amino acids and their derivatives, and saccharides, alongside the downregulation of lipids. This pattern suggests an adaptive metabolic state distinct from simple compositional differences reported previously. Importantly, this unique metabolic signature shows a closer association with the total antioxidant activity than with the contents of classical antioxidant components, hinting at a synergistic mechanism underlying its efficacy.

### 3.1. Exploring Potential Links Between Regional Conditions and Metabolic Patterns

The distinctive metabolite profile of Taizishen in Zherong may be linked to the specific soil conditions, climatic environment, and traditional processing methods of its production area. The patterns of metabolite accumulation observed in this study provide clues for understanding these potential connections. On one hand, the local acidic soil (pH 4.3–4.6) [28] could be a contributing factor. We observed a significant accumulation of amino acids and derivatives, such as γ-aminobutyric acid (GABA), in the Zherong samples. Notably, GABA is a well-known stress response metabolite in plants, and its biosynthesis is often activated under various stress conditions, including acid stress [29]. Therefore, the acidic soil environment presents a plausible and important factor potentially associated with the enrichment of GABA in Taizishen in Zherong.

In addition, the traditional “sun-drying” processing technique, in conjunction with the local high-humidity climate (average annual relative humidity approximately 81% [30]), may further shape its metabolic profile. Compared to other production areas that might employ artificial drying or are located in drier climates, the high humidity in Zherong results in a slower, more prolonged natural sun-drying process and extended dehydration time for the tuberous roots. This sustained dehydration stress likely induces the plant to synthesize more osmoprotectants for self-preservation. Previous studies have noted relatively higher levels of saccharides such as raffinose in Taizishen from Zherong [19]. The present study further confirms that the content of raffinose and trehalose is significantly higher in Zherong samples, and these saccharides are typical protective compounds synthesized by plants in response to osmotic stress [31,32]. This suggests that the combination of the “sun-drying” process and the specific climatic conditions may be the key driver behind the accumulation of functional saccharides in Taizishen in Zherong.

Simultaneously, the Zherong samples exhibited a highly significant co-accumulation of phenylalanine, fraxetin, and ethyl caffeate. This associated metabolite group holds promise as a key chemical marker for distinguishing the Zherong origin. Phenylalanine serves as the initial precursor of the phenylpropanoid pathway, whose downstream products include fraxetin (a coumarin) and ethyl caffeate (a phenolic ester) [33,34]. Therefore, their synchronized accumulation suggests that the specific conditions of the Zherong production area may influence the activity of this metabolic pathway. While abiotic stresses such as drought and pH variation are known to induce this pathway [35], the specific causal relationship between the observed metabolite enrichment and the environmental factors requires further verification through controlled experiments. The proposed chemical markers (e.g., fraxetin, ethyl caffeate) also need to be validated for their specificity and sensitivity using a larger-scale, more geographically diverse sample set.

### 3.2. Research Progress on Major Chemical Constituents and New Metabolomic Findings in P. heterophylla

To date, the diversity of reported saponins remains limited, with only a few compounds, such as Pseudostellarinoside A and Acutifolioside D, being frequently identified [36]. The TZS saponins have been shown to inhibit retinal cell apoptosis by suppressing oxidative stress and downregulating the expression of c-fos and Bax genes induced by retinal laser-induced injury [37].

In addition to saponins, earlier studies have reported relatively few flavonoid constituents in Taizishen. The flavonoids identified in *P. heterophylla* include: Acacetin, Luteolin, and Acacetin 7-O-β-D-glucopyranosyl(6→1)-α-L-rhamnopyranoside [38]. Among them, Acacetin and Luteolin are two flavonoids that have been relatively well-studied. Research indicates that they possess antioxidant, anti-inflammatory, neuroprotective, and potential anticancer activities. The effects of acacetin involve the modulation of multiple signaling pathways to inhibit inflammation and induce tumor cell apoptosis [39]. Luteolin primarily exerts immunomodulatory effects by regulating the functions of various immune cells, including T cells and macrophages [40], and can specifically inhibit the activation of Toll-like receptor 4 (TLR4) [41].

In the present study, more than 30 flavonoid-related metabolites were identified, including flavans, flavonoid glycosides, and O-methylated flavonoids. Among these, the content of the flavonoid compound Mundulone was significantly higher in the Zherong samples than in other major production areas. An in silico study using virtual screening and molecular dynamics simulations discovered that Mundulone can specifically bind to the ATP-binding site of Fibroblast Growth Factor Receptor 1 (FGFR1), demonstrating potential as an FGFR1 inhibitor [42]. Although the biological functions and mechanisms of flavonoids in Taizishen are not yet fully elucidated, our findings indicate a correlation between flavonoid content and antioxidant activity, suggesting that these components may contribute to the bioactive composition of Taizishen.

Heterophyllin represents another major class of chemical constituents in Taizishen, including heterophyllin A, B, C, and related compounds [43,44,45]. Among these, heterophyllin B has attracted considerable attention due to its diverse bioactivities, such as anti-inflammatory and anticancer properties [46,47,48]. Previous studies have indicated that the content of heterophyllin B was influenced by cultivation regions and agricultural practices. For instance, Taizishen cultivated in Zherong, Fujian Province, exhibited higher levels of Heterophyllin A and heterophyllin B compared with samples from Jurong, Jiangsu Province [19]. Consistent with these reports, both metabolomic and HPLC analyses in the present study revealed that the content of heterophyllin B in Taizishen from Zherong was higher than that in samples from other regions, except for Jurong.

Furthermore, a series of characteristic metabolites enriched in Taizishen in Zherong are associated with multiple potential pharmacological functions. For example, γ-aminobutyric acid is linked to neuromodulatory functions [49]; Fraxetin protects against rotenone-induced neurotoxicity in human neuroblastoma cells by enhancing cellular antioxidant defense (through GSH elevation) and inhibiting mitochondrial apoptosis pathways [50]; ethyl caffeate, a major active component of the traditional Chinese medicine compound “Feiyanning Formula” (FYN), has been shown to significantly delay or reverse acquired resistance to the third-generation EGFR-targeted drug osimertinib in non-small cell lung cancer [51]; and bruceine D is studied as a natural compound with anti-breast cancer metastasis activity [52]. The co-existence of these compounds with diverse biological activities in Taizishen in Zherong provides a potential chemical basis for its multifaceted traditional efficacy.

### 3.3. Antioxidant Activity May Arise from the Synergistic Effects of Multiple Classes of Metabolites

Taizishen has been reported to possess significant antioxidant activity [53] and is capable of effectively scavenging various free radicals, including DPPH·, ·OH, and O_2_^−^, thereby interrupting ROS-induced chain oxidation reactions and alleviating oxidative stress-related damage [54]. Moreover, Taizishen extracts significantly enhance the activities of antioxidant enzymes such as superoxide dismutase (SOD) and glutathione peroxidase (GSH-Px) while reducing malondialdehyde (MDA) levels, indicating a dual role in promoting endogenous antioxidant defense systems and inhibiting lipid peroxidation [55]. In the present study, correlation analysis revealed no significant association between antioxidant activity and saponin content, and only a weak correlation with total flavonoids, although previous studies have indicated that flavonoids generally possess antioxidant activity [56]. These findings suggest that the antioxidant effects of Taizishen are not attributable to a single class of compounds but rather result from synergistic interactions among multiple chemical constituents. Potential mechanisms underlying this synergy may include: (1) the simultaneous involvement of certain terpenoids, alkaloids, flavonoids, and other substances in free-radical scavenging [57]; and (2) the ability of specific amino acids such as phenylalanine (Phe) to enhance antioxidant metabolism by regulating the activity of antioxidant enzymes (e.g.,CAT, PAL) and glutathione (GSH) synthesis [58]. Studies have shown that amino acids are the main contributors to antioxidant activity in wheat- and rice-based gochujangs, and their correlation with antioxidant activity is stronger than that of flavonoids [59].

The untargeted metabolomics approach used in this study provides a new perspective for systematically elucidating the chemical basis and potential physiological mechanisms underlying the geo-authenticity of Taizishen. However, that metabolomic data reflect correlation rather than causation. The specific regulatory mechanisms linking the observed metabolic patterns to environmental factors (such as acidic soil and sun-drying processing), as well as the specificity of the screened differential metabolites (e.g., fraxetin, ethyl caffeate) as markers of geographical origin, still require verification through controlled experiments and validation using a larger-scale, more geographically diverse sample set. Moving forward, to achieve precise quality control and evaluation of Taizishen and to overcome the limitations of relying on a single omics approach, it is ultimately essential to establish a multidimensional quality evaluation model that integrates chemical markers, pharmacodynamic bioactivity data, genomic authentication, and key agronomic parameters. This will be crucial for ensuring the consistent quality and reliable efficacy of Pseudostellariae Radix medicinal materials and for enabling their precise application in clinical and health contexts.

## 4. Materials and Methods

### 4.1. Samples

The sample consists of *P. heterophylla* root tubers, harvested during the local traditional harvesting period (July to August) in 2022 and naturally sun-dried. Subsequently, the tubers were ground into powder, passed through a 40-mesh sieve, and stored sealed in double-layered ziplock bags protected from light at −20 °C. Detailed information about the ten samples collected from different production areas was provided in Table 1. All samples were taxonomically identified by Researcher Jingying Chen.

### 4.2. Chemical Determination

#### 4.2.1. Determination of Total Flavonoids

The determination of total flavonoid content was conducted in accordance with the Standard of the People’s Republic of China for Entry-Exit Inspection and Quarantine (SN/T 4592–2016, Determination of Total Flavonoids in Export Food) [60]. Rutin (, National Institutes for Food and Drug Control, Beijing, China, Batch No: 100080-201610,) was employed as the standard. After 1 mL, 2 mL, 3 mL, and 5 mL of rutin solution (1 mg/mL) were separately transferred into 50 mL volumetric flasks, anhydrous ethanol was added to adjust the volume to 15 mL. Subsequently, 1 mL of aluminum nitrate solution (100 g/L) and 1 mL of potassium acetate solution (98 g/L) were added, and the final volume was adjusted to 50 mL with distilled water. Then the reaction mixtures were incubated at room temperature for 1 h and the absorbance was measured at 420 nm. The calibration curve for rutin was established as y = 1.2659x + 0.0054 (R^2^ = 0.9992). For the preparation of the sample solution, 5 g of powdered sample was precisely weighed into a 100 mL Erlenmeyer flask, mixed with 30 mL of 100% ethanol, and fitted with a stopper. The total weight was accurately measured and recorded. The mixture was then ultrasonically extracted (power: 400 W, frequency: 40 kHz) at 30 °C for 60 min. After extraction, 100% ethanol was added to compensate for solvent loss and readjust the total weight to the initially recorded value. The mixture was centrifuged at 8000 rpm for 10 min, and the resulting supernatant was collected as the sample stock solution. For sample analysis, 5.0 mL of the sample stock solution was transferred into a 50 mL volumetric flask and treated following the same procedure as described for the standards. The total flavonoid content of the samples was calculated based on the rutin calibration curve.

#### 4.2.2. Determination of Total Saponins

Ginsenoside Rb1 (, National Institutes for Food and Drug Control, Beijing, China, Batch No: 110703-201530, 91.7%) was used as the standard, and 18 mg of ginsenoside Rb1 was dissolved in 25 mL of methanol to prepare the standard stock solution. Aliquots of the standard solution (0.1, 0.2, 0.3, 0.4, and 0.5 mL) were transferred into separate 10 mL test tubes, and the solvent was evaporated to dryness in a boiling water bath. Subsequently, 0.2 mL of 5% (*w*/*v*) vanillin in glacial acetic acid solution (freshly prepared before use) and 0.8 mL of perchloric acid were added. The reaction mixtures were incubated in a water bath at 60 °C for 15 min, after which 5 mL of glacial acetic acid was added. After cooling with running water, the absorbance was measured at 560 nm to obtain the standard curve (y = 2.7766x + 0.1144, R^2^ = 0.9993). For sample analysis, 2.0 g of powdered sample was extracted with 32 mL of 70% (*v*/*v*) ethanol using ultrasonic extraction (power: 400 W, frequency: 40 kHz) at 60 °C for 25 min. After cooling to room temperature, the extract was filtered under vacuum using a Buchner funnel. The filtrate was evaporated to dryness at 60 °C under a pressure of 170 MPa. The residue was then extracted twice with water-saturated n-butanol: first with 60 mL, followed by vigorous shaking and standing overnight, and then with 20 mL using gentle shaking. The combined organic extracts were evaporated to dryness under reduced pressure at 70 °C and 50 MPa. The resulting residue was dissolved in methanol and brought to a final volume of 25 mL, obtaining the sample saponin test solution. An aliquot of 0.1 mL of this solution was then subjected to the same colorimetric procedure as described for the standards, and the saponin content was calculated based on the calibration curve.

#### 4.2.3. Determination of Heterophyllin B

##### Sample Extraction

The powdered sample (2.0 g) was precisely weighed and mixed with 50 mL of methanol in a stoppered flask. The total weight was accurately recorded. The mixture was then extracted using ultrasonicate method at a power of 250 W and a frequency of 30 kHz for 45 min. After cooling, the total weight was measured again, and methanol was added to compensate for the weight loss. The extract was centrifuged at 8000 rpm for 5 min, and 25 mL of the supernatant was collected and transferred to a round-bottom flask. The solvent was subsequently evaporated to dryness by rotary evaporation at 40 °C. The residue was re-dissolved in 10 mL of methanol. After filtration through a 0.45 μm membrane filter, the final solution was used for HPLC analysis.

##### HPLC Detection

The HPLC analysis was performed on an Agilent 1260 high-performance liquid chromatography system (Agilent Technologies, Santa Clara, CA, USA) equipped with an Eclipse Plus C18 column (4.6 mm × 250 mm, 3.5 μm; Agilent Technologies) and the column temperature was 25 °C. The injection volume was 20 μL with a flow rate of 1 mL·min^−1^. The Mobile consisted of water (phase A) and acetonitrile (phase B). The gradient elution was as follows: 0–10 min, 2–10% B;10–40 min, 10–45% B; 40–45 min, 45–55% B; 45–50 min, 55–2% B; 50–55 min, 2% B, and the detection wavelength was set at 203 nm.

##### Standard Curve Preparation

Heterophyllin B (Purity ≥ 98%, Chengdu Aifa Biotechnology Co., Ltd., Chengdu, China, Batch No: AF22011101) was used as a standard and dissolved in methanol to prepare a stock solution with a concentration of 1 mg·mL^−1^. Then, the stock solution was serially diluted to concentrations of 0.01, 0.02, 0.03, 0.04, 0.05, and 0.10 mg/mL, and the corresponding absorption peak areas were measured. The standard curve was obtained as follows: y = 67.370x + 52.166 (R^2^ = 0.9993).

#### 4.2.4. Metabolomics Analysis

##### Sample Treatment

Approximately 100 mg of each sample was added to 1 mL of methanol-acetonitrile water solution (*v*/*v*, 2:2:1) and extracted using low-temperature ultrasonication for 30 min. After standing at −20 °C for 60 min, the extract was centrifuged at 14,000 *g* and 4 °C for 20 min. The supernatant was collected and vacuum dried, and then dissolved in 100 μL acetonitrile water solution (*v*/*v*, 1:1).

##### LC-MS/MS Detection

The chromatographic column was ACQUITY UPLC BEH Amide (1.7 μm, 2.1 mm × 100 mm) (Waters Corporation, Milford, MA, USA), and its temperature was 25 °C. The injection volume was 2 μL with a flow rate of 0.5 mL·min^−1^. Mobile phase A consisted of a water solution containing 25 mM ammonium acetate and 25 mM ammonium hydroxide, and mobile phase B was acetonitrile. The gradient elution program was as follows: 0–0.5 min, 95% B; 0.5–7 min, 95–65% B; 7–8 min, 65–40% B; 8–9 min, 40% B; 9–9.1 min, 40–95% B; 9.1–12 min, 95% B.

Mass spectrometry analysis was performed using a Triple TOF 6600 mass spectrometer (Sciex, Marlborough, MA, USA) under electrospray ionization (ESI) in both positive and negative ion modes. The nebulizer gas (Gas1) and auxiliary heating gas (Gas2) were set to 60 psi, and the curtain gas (CUR) was set to 30 psi. The ion source temperature was 600 °C, and the spray voltage (ISVF) was 5500 V in both positive and negative modes. The detection range for both the precursor (MS1) and product ions (MS2) was 60–1000 Da and 25–1000 Da, respectively.

##### Metabolomics Data Analysis

Raw data was converted to mzXML format using ProteoWizard software (version 2.0), and then the XCMS program (version 3.16.1) was used for peak alignment, retention time correction, and peak area extraction. Metabolites were identified by matching accurate mass (<25 ppm) and secondary spectrum matching, utilizing a self-written R package for peak identification. After peak detection, the data were filtered by removing features with >50% missing values within any experimental group, imputing remaining missing values with half the feature’s minimum value, and applying internal standard normalization. Features with a relative standard deviation (RSD) > 30% in quality control samples were excluded. Qualitative analysis was conducted using databases including KEGG, HMDB, CAS, Metlin, PubChem, and chEBL. Principal Component Analysis (PCA) and Orthogonal Partial Least Squares Discriminant Analysis (OPLS-DA) were performed using R software (version 4.2.0) with the MetaboAnalystR package (version 3.2.0). Specifically, PCA was first applied to assess the intrinsic data distribution and detect any outliers. Subsequently, to maximize group separation and identify the variables responsible for classification, supervised OPLS-DA was applied. The model’s goodness-of-fit (R2Y) and predictive ability (Q2) were calculated, and its robustness was validated through a 200-permutation test. Variable Importance in Projection (VIP) scores were extracted to evaluate each metabolite’s contribution to the model.

Differential metabolites were selected based on the following criteria: *t*-test (Student’s *t*-test) with a *p*-value < 0.05, fold change >1.5 or <0.67, and variable Importance in the Projection (VIP) score of the first principal component in the OPLS-DA model >1. Pathway analysis for these significant metabolites was conducted using the KEGG and MetaboAnalyst databases.

### 4.3. Antioxidant Ability Measurement

#### 4.3.1. Sample Treatment

Twenty grams of sample powder was ultrasonically extracted (power: 400 W, frequency: 40 kHz) at 30 °C with 100 mL of 70% ethanol for 45 min. The extract was evaporated at 50 °C, freeze-dried, and the residue was dissolved in ddH_2_O to prepare a solution with a concentration of 10 mg/mL for subsequent experiments.

#### 4.3.2. DPPH Free Radical Scavenging Rate

To evaluate the DPPH radical scavenging ability, 0.2 mL of the sample solution (10 mg/mL) was mixed with 0.9 mL of anhydrous ethanol and 0.9 mL of a *2,2-Diphenyl-1-picrylhydrazyl* (DPPH) solution in the DPPH assay kit (Boer, Shanghai, China). The absorbance was measured at 515 nm. A blank control was prepared by replacing the sample solution with 0.2 mL of ddH_2_O. The DPPH radical scavenging rate was calculated using the following formula: Scavenging Rate (%) = [(OD*blank* − OD*sample*)/OD*blank*] × 100%.

#### 4.3.3. ABTS Free Radical Scavenging Rate

Total antioxidant capacity assay kit with a rapid *2,2′-Azino-bis(3-ethylbenzothiazoline-6-sulfonic acid)* (ABTS) method (Beyotime, Shanghai, China) was used for the ABTS free radical scavenging rate determination. Trolox standard solutions (10 mM) were gradient diluted to the concentrations of 0.1, 0.2, 0.3, 0.4, 0.5, and 0.6 mM. The absorbance was measured at 414 nm to construct the standard curve (y = 1.225x + 0.0235, R^2^ = 0.9935). The Trolox-equivalent antioxidant ability was calculated using the formula: Trolox Equivalent Antioxidant Capacity = (OD*sample* − 0.0235)/1.225. For the assay, 20 μL of peroxidase solution, 10 μL of the sample, and 170 μL of ABTS solution were mixed. After incubation at room temperature for 6 min, the absorbance at 414 nm was measured.

#### 4.3.4. Iron Ion Reducing Ability

Total antioxidant capacity assay kit with *Ferric Reducing Antioxidant Power* (FRAP) method (Beyotime, Shanghai, China) was used for the iron ion reducing ability determination. FeSO_4_·7H_2_O solution (100 mM) from the reagent kit was prepared, and subsequently diluted to the concentrations of 0.1, 0.2, 0.3, 0.4, 0.5, and 0.6 mM. Absorbance was measured at 593 nm to generate the standard curve (y = 0.3086x + 0.0637, R^2^ = 0.9927). The antioxidant capacity was calculated using the following formula: FeSO_4_ Equivalent Antioxidant Capacity = (A593 sample − 0.0637)/0.3086. For the assay, 5 μL of the sample was added to 180 μL of FRAP working solution, and the mixture was incubated at 37 °C for 3 to 5 min. Absorbance at 593 nm was measured, and the iron ion reducing ability was determined based on the standard curve.

## 5. Conclusions

This study systematically compared the chemical composition, metabolic profiles, and antioxidant activities of *P. heterophylla* (Taizishen) from different MP and NP areas across different regions in China. The results confirmed significant regional disparities in both chemical constituents and bioactivity. Notably, samples from Jurong and Zherong contained higher levels of key bioactive components (e.g., total saponins, total flavonoids, and heterophyllin B). More importantly, untargeted metabolomics revealed a distinct metabolic signature in Taizishen in Zherong, characterized by the coordinated accumulation of amino acids, sugars, and specific secondary metabolites (e.g., fraxetin, ethyl caffeate), coupled with a reduction in lipids. Antioxidant activity varied substantially among producing areas, and was correlated with multiple metabolite classes rather than solely with flavonoids or saponins, suggesting a synergistic contribution to its efficacy. In summary, geographical origin profoundly shapes the chemoprofile and bioactivity of Taizishen. These findings underscore the necessity of developing a comprehensive, metabolomics-informed quality evaluation system to ensure the consistency and clinical efficacy of this medicinal material.

## Figures and Tables

**Table 1:** Sample information.

Sample Name	Producing Areas	Latitude and Longitude	Altitude (m)
F1	Fuan, Fujian (NP area)	27°08′ N, 119°38′ E	70
F2	Zherong, Fujian (MP area)	27°16′ N, 119°50′ E	900
A1	Guangde, Anhui (NP area)	30°56′ N, 119°21′ E	80
A2	Xuanchen, Anhui (MP area)	30°51′ N, 118°50′ E	70
G1	Shibin, Guizhou (MP area)	27°8′ N, 107°56′ E	900
G2	Duyun, Guizhou (NP area)	26°13′ N,107°38′ E	830
J1	Jurong, Jiangsu (MP area)	31°66′ N, 119°23′ E	30
J2	Ganyu, Jiangsu (NP area)	34°93′ N, 119°03′ E	100
S1	Changyi, Shandong (NP area)	36°40′ N, 117°28′ E	80
S2	Linmu, Shandong (MP area)	34°72′ N, 118°76′ E	50

## Data Availability

The original contributions presented in this study are included in the article/Supplementary material. Further inquiries can be directed to the corresponding author(s).

## Supplementary Materials

The following supporting information can be downloaded at: https://www.mdpi.com/article/10.3390/ijms27073139/s1.
