# Supplementary material for: Differences in Chemical Components and Antioxidant Ability Analysis of Pseudostellaria heterophylla from Multiple Origins
Source: Int J Mol Sci. 2026 Mar 30;27(7):3139. doi: 10.3390/ijms27073139 (PMC13074024; doi:10.3390/ijms27073139)
Supplement: Supplementary file 1 [file ijms-27-03139-s001.zip › Supplementary files/Supplementary Figure S1 OPLS-DA analysis of different production areas.pdf]

Supplementary Figure S1

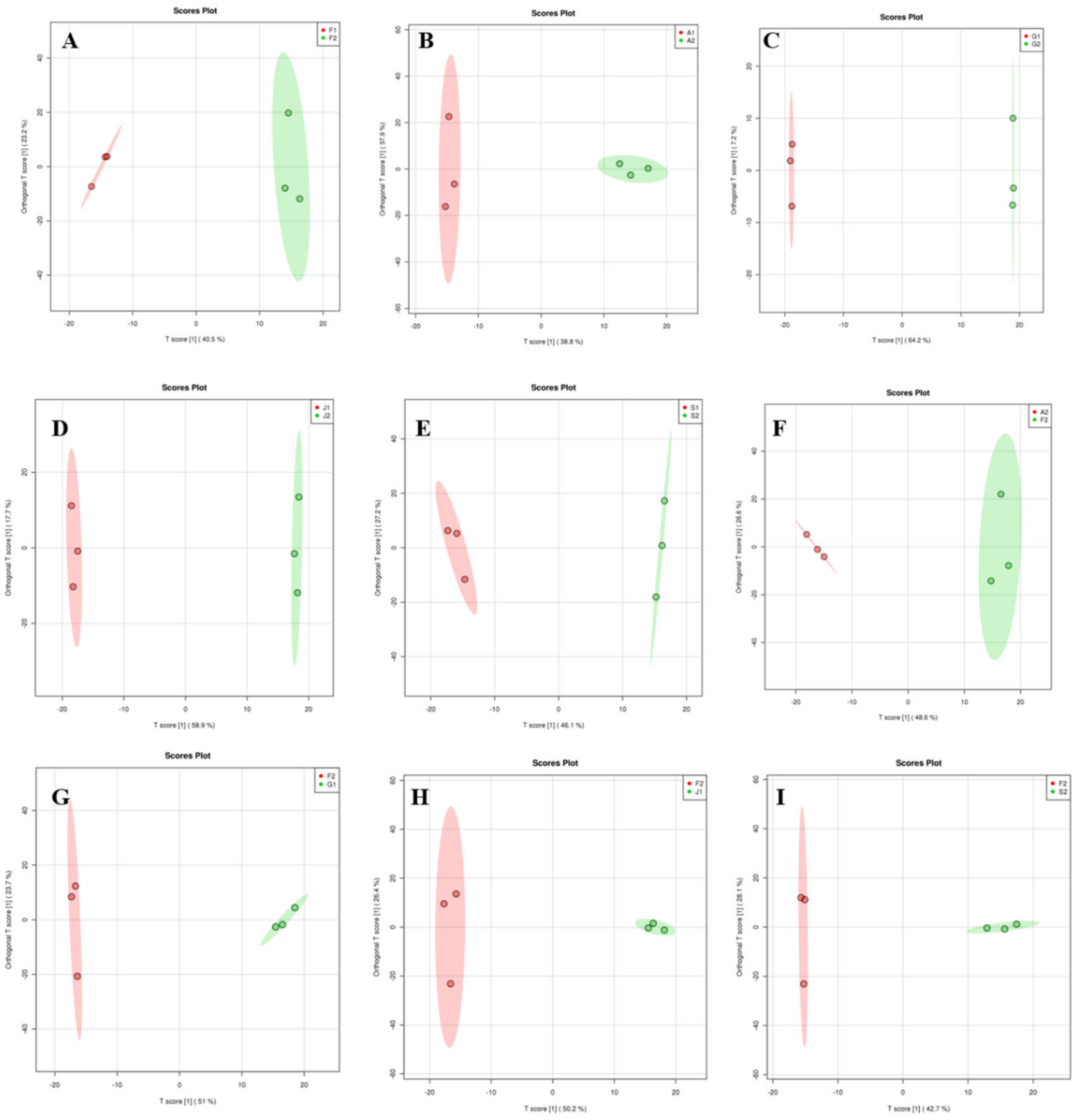

Supplementary Figure S1: OPLS-DA analysis of different production areas

A: F1_vs_F2, B: A1_vs_A2, C: G2_vs_G1, D: J2_vs_J1, E: S1_vs_S2, F: A2_vs_F2, G: G1_vs_F2, H: J1_vs_F2, I: S2_vs_F2

Plots A–I represent the following pairwise comparisons:

(A) F1 (Fuan, Fujian, NP) vs. F2 (Zherong, Fujian, MP)

(B) A1 (Guangde, Anhui, NP) vs. A2 (Xuancheng, Anhui, MP)

(C) G2 (Duyun, Guizhou, NP) vs. G1 (Shibin, Guizhou, MP)

(D) J2 (Ganyu, Jiangsu, NP) vs. J1 (Jurong, Jiangsu, MP)

(E) S1 (Changyi, Shandong, NP) vs. S2 (Linmu, Shandong, MP)

(F) A2 (Xuancheng, Anhui, MP) vs. F2 (Zherong, Fujian, MP)

(G) G1 (Shibin, Guizhou, MP) vs. F2 (Zherong, Fujian, MP)

(H) J1 (Jurong, Jiangsu, MP) vs. F2 (Zherong, Fujian, MP)

(I) S2 (Linmu, Shandong, MP) vs. F2 (Zherong, Fujian, MP)

(MP: main producing area; NP: non-main producing area)
